# Hydrogen Sulfide Attenuates High Glucose-Induced Human Retinal Pigment Epithelial Cell Inflammation by Inhibiting ROS Formation and NLRP3 Inflammasome Activation

**DOI:** 10.1155/2019/8908960

**Published:** 2019-04-24

**Authors:** Peng Wang, Fei Chen, Wenyan Wang, Xue-Dong Zhang

**Affiliations:** The First Affiliated Hospital of Chongqing Medical University, Chongqing Key Laboratory of Ophthalmology and Chongqing Eye Institute, Chongqing, China

## Abstract

Hydrogen sulfide (H_2_S) has been shown to protect against oxidative stress injury and inflammation in various high glucose-induced insult models. However, it remains unknown whether H_2_S protects human retinal pigment epithelial cells (RPE cells) from high glucose-induced damage. In the current study, cell viability, proinflammatory cytokines, ROS, and inflammasome markers were compared in a low glucose- and high glucose-induced cell culture system. The antioxidant N-acetylcysteine (NAC), NLRP3 siRNA, and NaHS were used to test RPE cell responses. The results demonstrate that compared with the low-glucose culture, high glucose triggered higher cell death and increased IL-18 and IL-1*β* mRNA expression and protein production. Furthermore, high glucose increased the mRNA expression levels of NLRP3, ACS, and caspase-1. Notably, NAC, a ROS scavenger, could attenuate high glucose-induced ROS formation and IL-18 and IL-1*β* mRNA and protein expression and block inflammasome activation. Silencing the NLRP3 gene expression also abolished IL-18 and IL-1*β* mRNA and protein expression. Intrudingly, H_2_S could ameliorate high glucose-induced ROS formation, IL-18 and IL-1*β* expression, and inflammasome activation. Taken together, the findings of the present study have demonstrated that H_2_S protects cultured RPE cells from high glucose-induced damage through inhibiting ROS formation and NLRP3 inflammasome activation. It might suggest that H_2_S represents a potential therapeutic target for the treatment of diabetic retinopathy.

## 1. Introduction

Diabetic retinopathy (DR) is a common complication of diabetes and is also the leading cause of visual impairment and blindness [1]. It has been well recognized that hyperglycemia plays a pivotal role in the pathogenesis of DR. The chronic hyperglycemic environment damages not only the retinal vasculature but also other types of cells in the retina, such as retinal pigment epithelial (RPE) cells, which are a crucial component of the blood-retinal barrier and a key element in maintaining the proper function of the visual system [1, 2].

DR has been caused by a combination of multiple factors, although the etiology and progression of DR remain poorly understood. Several mechanisms have been found to be closely associated with DR. These include the activated polyol pathway and protein kinase C (PKC), increased expression of various growth factors, vascular endothelial growth factor (VEGF) and insulin-like growth factor-1 (IGF-1), and hemodynamic changes and the formation of advanced glycation end products (AGEs) [3]. Furthermore, it has also been well documented that increased oxidative stress, the activation of the renin-angiotensin-aldosterone system (RAAS), and subclinical inflammation and capillary occlusion also play important roles in the pathogenesis of DR [3, 4].

Recent studies have indicated that the pathogenic effect triggered by oxidative stress was induced *via* the promotion of inflammatory response and apoptosis through the activation of the downstream signaling pathway [5–7]. Furthermore, moderate levels of reactive oxidative species (ROS) play an important role in the immune response to foreign pathogens, while high levels of ROS often lead to pathogenesis and disease. In diabetes mellitus, oxidative stress can be activated through the polyol pathway, PKC, AGEs, amidohexose, and other metabolic pathways [8, 9], and such extensive levels of ROS could subsequently lead to neovascularization and the activation of inflammatory cytokines [10], which in turn accelerates DR progression.

The NLRP3 inflammasome, which is a complex formed by NLRP3, ASC, and caspase-1, is a key element in inflammatory immune response *via* caspase-1 activation and proinflammatory cytokine IL-1*β* and IL-18 secretion after activation [11]. Recent studies have indicated that mitochondrial ROS generation is closely linked with the activation of NLRP3, suggesting that the NLRP3 inflammasome is a critical sensor of mitochondrial dysfunction, and that this might well explain why many metabolic disorders are associated with mitochondrial damage [12]. NLRP3 has also been reported to play an important role in diabetes and other metabolic diseases [13]. A recent study revealed that high glucose could stimulate ROS formation and NLRP3 inflammasome activation and eventually increase cell apoptosis and retinal vascular permeability in human retinal microvascular endothelial cells (HRMECs) and the retina of diabetic rats [14]. In addition, blocking NLRP3 inflammasome activation by siRNA silencing could attenuate these effects [14].

Hydrogen sulfide (H_2_S) is known to be synthesized intracellularly from cysteine by cystathionine gamma-lyase (CSE) and obtained from other naturally occurring enzymes. Furthermore, H_2_S has been reported to play an important role in the pathophysiology of the nervous system, circulatory system, immune system, and endocrine system [15]. Growing evidence suggests that H_2_S has a protective role against various inflammatory-stimulated injuries in tissues, such as those from the heart, liver, and kidneys [16–18]. Furthermore, H_2_S mainly works as an antioxidant [19]. On the one hand, it has been reported that the addition of H_2_S could increase the reduced form of intracellular glutathione (GSH) in a monocyte cell model [20]. On the other hand, H_2_S has been reported to directly scavenge superoxide anions, hydrogen peroxide (H_2_O_2_), and peroxynitrite [21]. Furthermore, H_2_S and its endogenous enzymes have been considered to play a pivotal role in the pathogenesis of diabetes and its complications, since studies have confirmed that H_2_S levels decrease in diabetic patients and diabetic rats [22, 23] and, in particular, tissues with diabetic endothelial dysfunction, nephropathy, and cardiomyopathy [24, 25]. Moreover, it has been shown that retinal tissue can also produce H_2_S, which might be involved in the pathogenesis of retinal degeneration and retinal ischemia-reperfusion injury [26]. Based on these findings, the present study intended to investigate whether exogenous H_2_S could protect human retinal pigment epithelial (ARPE-19) cells against high glucose-induced damage and its role in anti-inflammation, especially the NLRP3 inflammasome.

## 2. Materials and Methods

### 2.1. Materials

Sodium hydrosulfide (NaHS) was purchased from Chuandong Chemical Group Co. Ltd. (Chongqing, China). N-Acetyl-L-cysteine (NAC) and 2,7-dichlorofluorescein diacetate (DCFH-DA) were manufactured by Sigma-Aldrich (St. Louis, MO, USA). Cell Counter Kit-8 (CCK-8) was purchased from Dojindo (Kumamoto, Japan). Dulbecco's modified Eagle's medium F12 (DMEM/F12) and fetal bovine serum (FBS) were purchased from Gibco-BRL (Carlsbad, CA). TRIzol RNA isolation reagent was purchased from Invitrogen Life Technologies (New York, New York, USA). The first strand cDNA synthesis kit and SYBR green reagents were purchased from Takara (Dalian, China). siRNA of NLRP3 was obtained from Hanbio Biotechnology Co. Ltd. (Shanghai, China).

### 2.2. Cell Culture

Human retinal pigment epithelium cell line ARPE-19 was obtained from the American Type Culture Collection (ATCC). Cells were cultured in (1 : 1) mixed Dulbecco's modified Eagle's medium : nutrient mixture F12 (DMEM/F12), supplemented with 10% heat-inactivated fetal bovine serum (FBS) (Gibco, Grand Island, NY, USA) and antibiotics (100 mg/mL of streptomycin and 100 U/mL of penicillin). Then, cells were cultured at 37°C in a humidified chamber with 5% CO_2_ and passaged every 5-7 days. Trypsin-EDTA solution (diluted from 1 : 3 to 1 : 4) was used to dissociate cells from the culture flasks (Corning, Lowell, MA, USA) after cells reached full confluency. Six testing cell culture groups were established: (1) LG: low-glucose group (5.5 mM of glucose), (2) HG: high-glucose group (25 mM of glucose), (3) NAC: high-glucose+NAC group (2.5 mM of NAC in 25 mM of glucose medium), (4) NLRP3 siRNA: high-glucose+NLRP3 siRNA group (cells were transfected with NLRP3 siRNA), (5) control siRNA: high-glucose+scrabble siRNA group (cells were transfected with scrabble siRNA), and (6) H_2_S: high-glucose+H_2_S group (200 *μ*M of NaHS in 25 mM of glucose medium). Cells in each group were induced for 48 hours before further experimental tests.

### 2.3. Cell Viability Assay

ARPE-19 cells were cultured in 96-well plates with or without the various treatments. Then, a 10 *μ*L CCK-8 solution (1 : 10 dilution) was added to each well and incubated for an additional two hours. The absorbance at 450 nm was recorded using a microplate reader, and cell viability was calculated according to the manufacturer's instructions.

### 2.4. ELISA

IL-18 and IL-1*β* levels from the supernatant of homogenized cultured cells were measured by ELISA. An IL-1*β* ELISA kit (eBioscience; Ref: BMS630, Lot: 87225015) and an IL-18 ELISA kit (Novex; Ref: KRC2341, Lot: 130401/A) were used for the analysis. Each sample was tested at least three times.

### 2.5. Measurement of Intracellular ROS

After various treatments, 10 *μ*M of DCFH-DA was added to each well and incubated at 37°C for 30 minutes. Then, cells were washed with PBS for three times. Next, cells were resuspended in PBS at 1 × 10^6^ cells/mL, and the fluorescence of the oxidized dichlorofluorescein (DCF) product was measured *via* fluorescence-activated cell sorting (FACS) flow cytometry at an excitation of 488 nm and an emission of 525 nm. Untreated cells served as controls. These results were expressed as the fluorescence intensity of DCF relative to control.

### 2.6. Real-Time Polymerase Chain Reaction (PCR)

Total RNA was extracted and isolated from ARPE-19 cells using the TRIzol reagent (Life Technologies, New York, USA), according to the manufacturer's instructions. Quantitative PCR was performed using the Applied Biosystems 7500 Fast Real-Time PCR System (Foster City, CA, USA).

The primers (Table 1) were designed and synthesized by Sangon Biotech (Shanghai, China). Real-time PCR was performed using the SYBR Green master mix, according to the standard thermocycler conditions. The target gene expression was quantified relative to the housekeeping gene *β*-actin *via* the optimized comparative 2^-ΔΔCT^ value method, as reported by a previous study [27].

### 2.7. Antioxidant Intervention

For the antioxidant-related test, NAC at a concentration of 2.5 mM was used to treat ARPE-19 cells cultured under low- and high-glucose conditions for 48 hours. Then, cells were collected and subjected to RT-PCR and ELISA.

### 2.8. Gene Silencing Assay

NLRP3 siRNA with its sequence of 5′-CACGTGTTTCGAATCCCACTGTGAT-3′ and scrabble siRNA with its sequence of 5′-CATGGATTGGTGAACAGCCACCTCA-3′ were obtained from Hanbio Biotechnology Co. Ltd. (Shanghai, China). The siRNA was delivered using Lipofectamine 2000 (Life Technologies, California, USA), according to the manufacturer's instruction. After siRNA transfection, cells were cultured for another 48 hours, and the supernatant was collected for ELISA.

### 2.9. Statistical Analysis

All data were expressed as mean ± standard error (SE). One-way analysis of variance (ANOVA) was used to determine the significance between groups using the SPSS software (version 17.0, Chicago, IL, USA). A *P* value < 0.05 was considered statistically significant.

## 3. Results

### 3.1. High Glucose Decreases ARPE-19 Cell Viability and Induces Inflammatory Cytokine Expression

**Figure 1:**
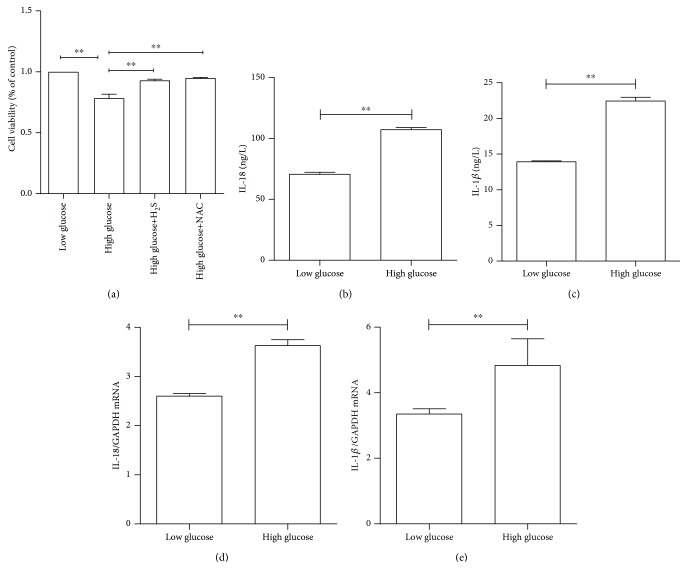
The high-glucose condition decreased cell viability and induced proinflammatory cytokine IL-18 and IL-1*β* production in ARPE-19 cells. ARPE-19 cells were treated with high or low glucose for 48 hours. (a) Cell viability determined by the CCK-8 assay in the low-glucose group, high-glucose group, high-glucose+H_2_S group, and high-glucose+NAC group. (b) Protein levels of IL-18 detected by ELISA. (c) Protein levels of IL-1*β* detected by ELISA. (d) The mRNA expression levels of IL-18 determined by RT-PCR. (e) IL-1*β* mRNA levels measured by RT-PCR. Data were presented as the mean ± standard error (SE) of three independent experiments. ^∗^*P* < 0.05 and ^∗∗^*P* < 0.01, compared between the low-glucose and high-glucose groups (low glucose: 5.5 mM, high glucose: 25 mM).

To investigate the effect of the cell viability of RPE cells, human retinal pigment epithelial (ARPE-19) cells were cultured in medium with low glucose (5.5 mM) and high glucose (25 mM) for 48 hours, respectively. Cell viability was determined by the CCK-8 assay. As shown in Figure 1(a), a significantly reduced cell viability was observed in the 25 mM glucose culture condition, when compared with the 5.5 mM glucose medium (*P* < 0.01), which is consistent with a previous study conducted by the investigators [28]. But as shown in Figure 1(a), the NaHS and NAC could significantly attenuate high glucose-induced reduction of cell viability.

To further understand the cell responses under high-glucose stimulation, the intracellular production of two inflammatory cytokines IL-18 and IL-1*β* was measured by ELISA. As illustrated in Figures 1(b) and 1(c) and compared with the low-glucose culture condition, high glucose triggered an approximately 65% and 72% increase in IL-18 and IL-1*β* production in ARPE-19 cells, respectively (*P* < 0.01). Furthermore, the mRNA levels of IL-18 and IL-1*β* in ARPE-19 cells were also measured under high- vs. low-glucose culture conditions. As shown in Figures 1(d) and 1(e), similar results were found in mRNA expression relative to protein production.

### 3.2. High Glucose Increases Intracellular ROS Formation and Activates the NLRP3 Inflammasome in ARPE-19 Cells

**Figure 2:**
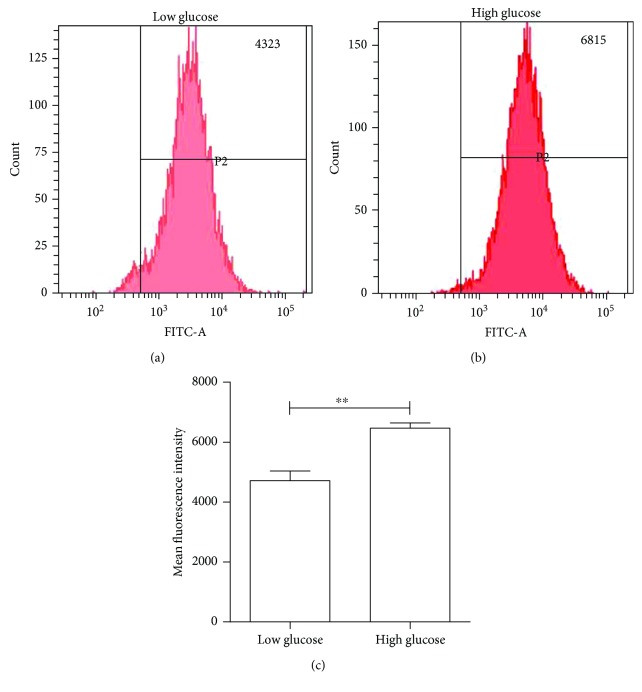
The high-glucose condition increased intracellular ROS formation in ARPE-19 cells. ARPE-19 cells were treated with high or low glucose for 48 hours. ROS production was measured by FCM. (a) FACS results of low-glucose culture condition. (b) FAC results of high-glucose culture condition. (c) A bar graph of average results obtained from three individual experiments. Data were presented as the mean ± standard error (SE) of three independent experiments. ^∗^*P* < 0.05 and ^∗∗^*P* < 0.01, compared between the low-glucose and high-glucose groups (low glucose: 5.5 mM, high glucose: 25 mM).

The increase in proinflammatory cytokine formation by high glucose inspired the investigators to determine the ROS production and inflammasome activation in RPE cells. To determine the ROS, cells were labeled with a fluorescence marker DCFH-DA, and the redox sensor and DCF fluorescence would form once DCFH-DA becomes intracellularly oxidized. Then, cells were quantitatively monitored by FACS flow cytometry. As indicated in Figures 2(a) and 2(b), significant levels of ROS were formed in high glucose-treated ARPE-19 cells, compared with low-glucose culture conditions, for 48 hours. The quantitative data is shown in Figure 2(c), and it could be observed that ROS increased by approximately 40% in high glucose-stimulated cells vs. low-glucose conditions.

**Figure 3:**
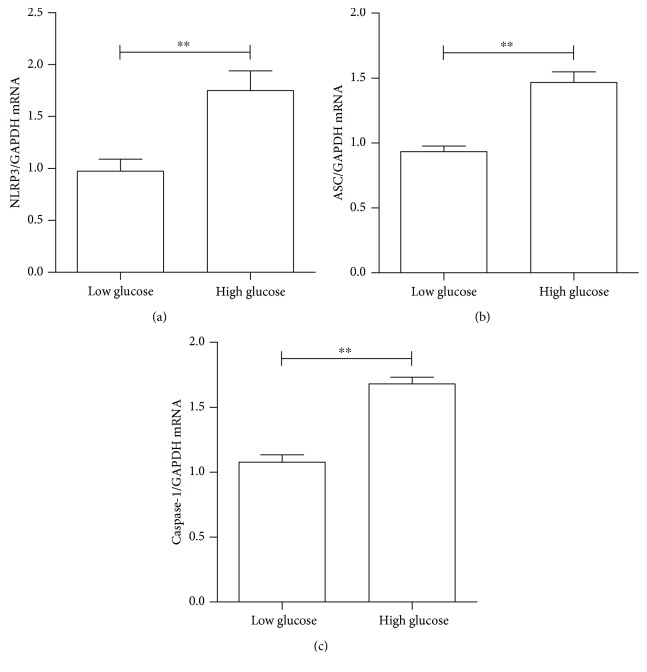
The high glucose-induced activation of the inflammasome in ARPE-19 cells. The mRNA expression of NLRP3, ASC, and caspase-1 in ARPE-19 cells after low-glucose and high-glucose culture for 48 hours was determined by RT-PCR. (a) NLRP3 mRNA levels were relative to GAPDH. (b) ASC mRNA levels were relative to GAPDH. (c) Caspase-1 mRNA levels were relative to GAPDH. Data were presented as the mean ± standard error (SE) of three independent experiments. ^∗^*P* < 0.05 and ^∗∗^*P* < 0.01, compared between the low-glucose and high-glucose groups (low glucose: 5.5 mM, high glucose: 25 mM).

To investigate whether inflammasome was activated in ARPE-19 cells by high-glucose culture conditions, the mRNA levels of inflammasome marker genes, such as *NLRP3*, *ASC*, and *caspase-1*, were determined. As shown in Figures 3(a)–3(c), NLRP3 mRNA levels increased by over 85%, ASC mRNA levels increased by over 65%, and caspase-1 mRNA levels increased by over 60% in 48 hours under high-glucose culture conditions, when compared with the low-glucose culture medium.

### 3.3. N-Acetylcysteine Attenuates High Glucose-Induced Inflammatory Response in RPE Cells

**Figure 4:**
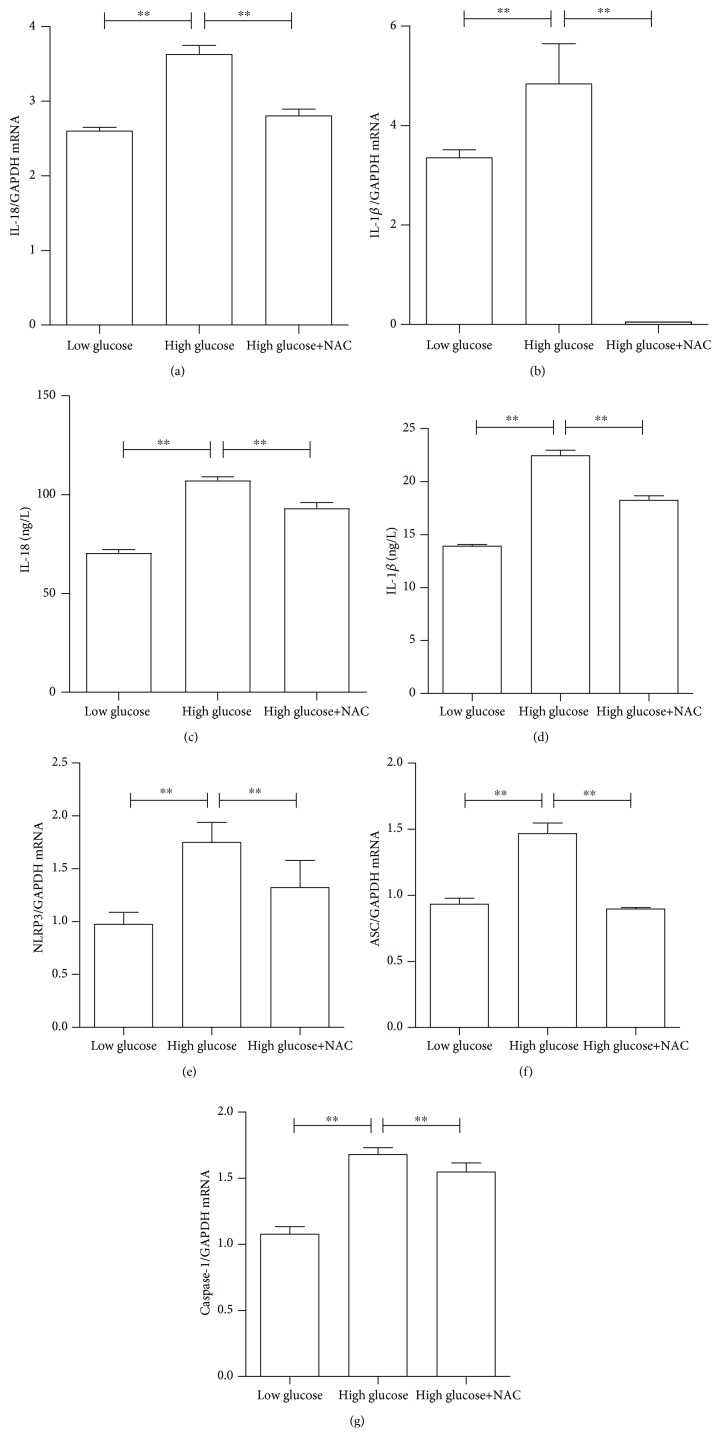
N-Acetylcysteine (NAC) attenuated intracellular ROS formation and inflammatory response. The inhibition of ROS by NAC decreased the gene expression of NLRP3, ASC, caspase-1, IL-18, and IL-1*β* and the protein expression of IL-18 and IL-1*β* in ARPE-19 cells. ARPE-19 cells were cultured under low or high glucose with or without 2.5 mM of NAC for 48 hours. The mRNA and protein expression levels of proinflammatory cytokines IL-18 and IL-1*β* and inflammasome activation markers NLRP3, ACS, and caspase-1 were determined, accordingly. (a) IL-18 mRNA; (b) IL-1*β* mRNA; (c) intracellular IL-18 protein levels; (d) intracellular IL-1*β* protein levels; (e) NLRP3 mRNA; (f) ASC mRNA; (g) caspase-1 mRNA. Data were presented as the mean ± standard error (SE) of three independent experiments. ^∗^*P* < 0.05 and ^∗∗^*P* < 0.01, compared between the high-glucose and high-glucose+NAC groups (low glucose: 5.5 mM, high glucose: 25 mM).

To verify the role of ROS in stimulating proinflammatory cytokine production and inflammasome activation, ARPE-19 cells were treated with or without 2.5 mM of N-acetylcysteine (NAC), which is an antioxidant, combined with high- or low-glucose culture medium for 48 hours. As shown in Figures 4(a) and 4(b), 2.5 mM of NAC coculture could significantly attenuate the mRNA expression level of high glucose-induced proinflammatory cytokines IL-18 and IL-1*β*, particularly IL-1*β*, which was almost entirely abolished by NAC. At the protein level (Figures 4(c) and 4(d)), NAC could significantly block IL-18 and IL-1*β* production (*P* < 0.01). Similarly, 2.5 mM of NAC could also significantly ameliorate high glucose-induced inflammasome activation. As presented in Figures 4(e)–4(g), NAC could almost abolish the high glucose-induced mRNA expression of both NLRP3 and ASC and significantly attenuate caspase-1 mRNA expression.

### 3.4. The Knockdown of NLRP3 Expression Attenuates High Glucose-Induced RPE Cell Inflammatory Response in ARPE-19 Cells

**Figure 5:**
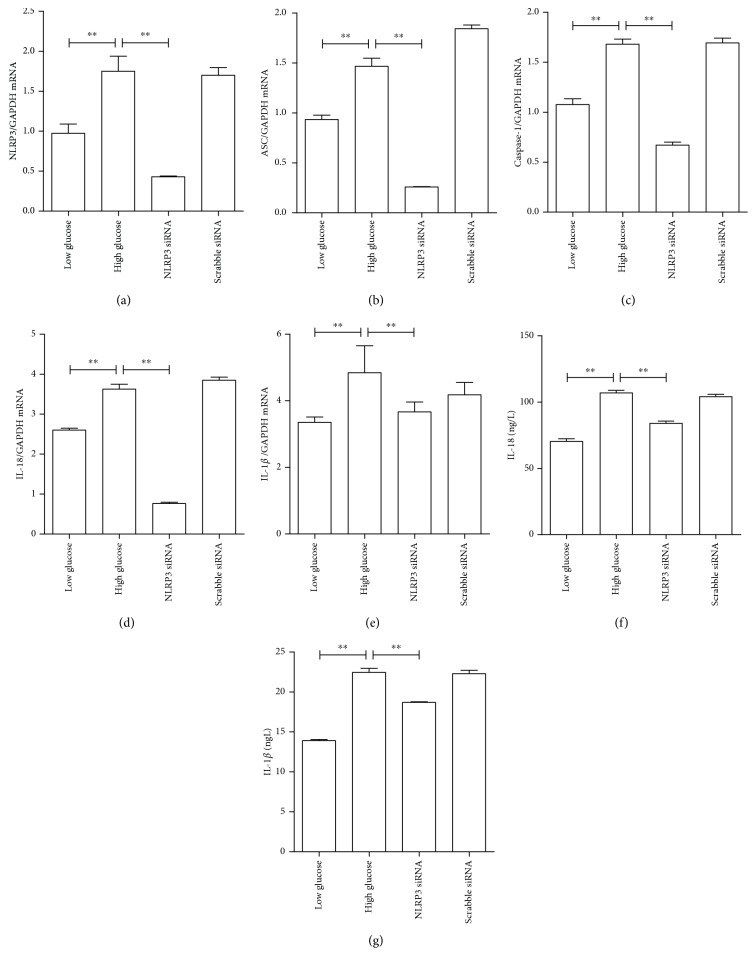
The knockdown of NLRP3 gene expression ameliorated high glucose-induced proinflammatory cytokine production and inflammasome activation. The gene expression levels of IL-18 and IL-1*β* and inflammasome markers NLRP3, ASC, and caspase 1 were measured in ARPE-19 cells with either NLRP3-siRNA or scrabbling siRNA transfection in low- and high-glucose culture for 48 hours. (a) NLRP3 mRNA; (b) ASC mRNA; (c) caspase-1 mRNA; (d) IL-18 mRNA; (e) IL-1*β* mRNA; (f) intracellular IL-18 protein levels; (g) intracellular IL-1*β* protein levels. Data were presented as the mean ± standard error (SE) of three independent experiments. ^∗^*P* < 0.05 and ^∗∗^*P* < 0.01, compared between the high-glucose and high-glucose+NLRP3 siRNA groups. There was no statistical significance between the glucose group and the NLRP3 siRNA group (low glucose: 5.5 mM, high glucose: 25 mM).

Next, the association between inflammasome activation and proinflammatory cytokine expression and production was further tested. To do this, the siRNA knockdown approach was used to reduce NLRP3 expression, and its effects on IL-18 and IL-1*β* gene expression and protein formation were tested. As shown in Figure 5(a), the specific siRNA that targeted NLRP3 significantly silenced NLRP3 expression, as determined by mRNA levels, but not by scrabbling siRNA. As shown in Figures 5(b) and 5(c), as expected, both ASC and caspase-1 mRNA expression levels were significantly reduced in high glucose-induced ARPE-19 cells after silencing NLRP3 gene expression. In addition, following siRNA-based NLRP3 gene silencing, it was observed that the mRNA expression of both IL-18 (Figure 5(d)) and IL-1*β* (Figure 5(e)) and the protein production of IL-18 (Figure 5(f)) and IL-1*β* (Figure 5(g)) were significantly reduced in high glucose-treated conditions, compared with scrabble siRNA-treated cells.

### 3.5. H_2_S Decreases High Glucose-Induced ROS Production, NLRP3 Inflammasome Activation, and Inflammatory Cytokine Production in ARPE-19 Cells

**Figure 6:**
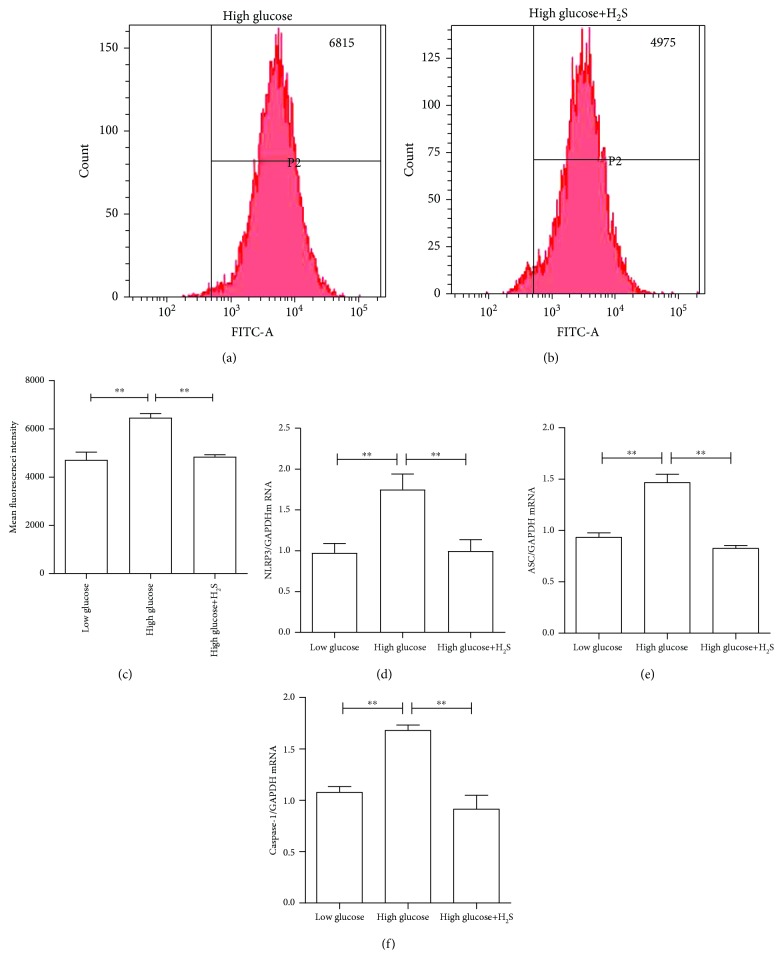
H_2_S decreased high glucose-induced ROS production and inflammatory response in RPE cells. Intracellular ROS formation and inflammasome marker NLRP3, ASC, and caspase-1 expression were determined in ARPE-19 cells after 48 hours of culture under low and high glucose, with or without 200 *μ*M of NaHS pretreatment. (a) The ROS production indicated by FACs in high-glucose culture; (b) the ROS production indicated by FACs in high-glucose+NaHS culture; (c) a bar graph of average results from the three individual experiments; (d) NLRP3 mRNA; (e) ASC mRNA; (f) caspase-1 mRNA. Data were presented as the mean ± standard error (SE) of three independent experiments. ^∗^*P* < 0.05 and ^∗∗^*P* < 0.01, compared between the high-glucose and high-glucose+H_2_S groups (low glucose: 5.5 mM, high glucose: 25 mM).

**Figure 7:**
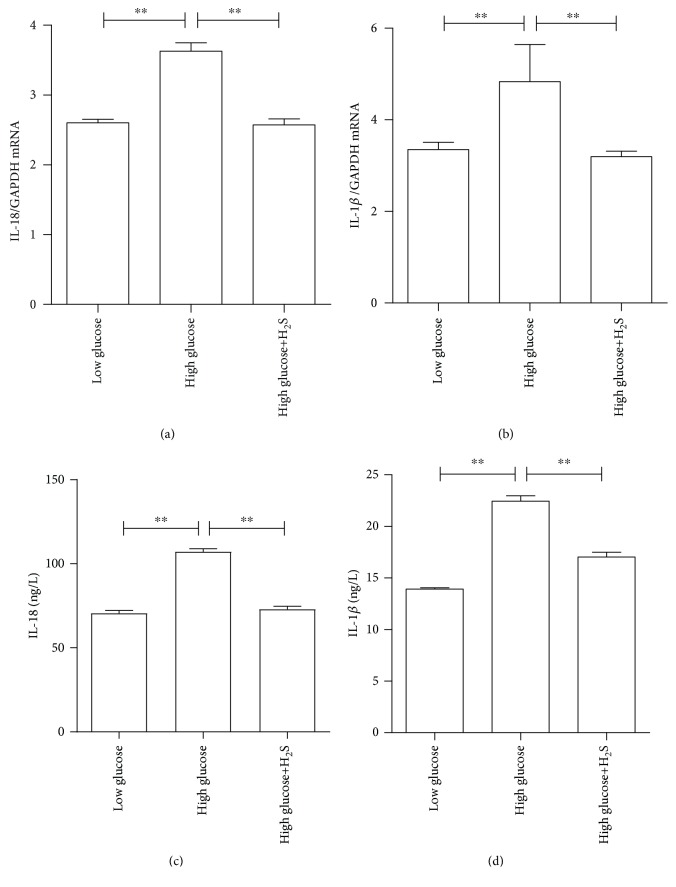
H_2_S decreased high glucose-induced proinflammatory cytokine IL-18 and IL-1*β* production in RPE cells. ARPE-19 cells were cultured in high glucose with or without 200 *μ*M of NaHS pretreatment. The mRNA and intracellular protein expression levels were measured, accordingly. (a) IL-18 mRNA; (b) IL-1*β* mRNA; (c) intracellular IL-18 protein levels; (d) intracellular IL-1*β* protein levels. Data were presented as the mean ± standard error (SE) of three independent experiments. ^∗^*P* < 0.05 and ^∗∗^*P* < 0.01, compared between the low-glucose and high-glucose groups (low glucose: 5.5 mM, high glucose: 25 mM).

Finally, the effect of H_2_S on high glucose-induced ROS production was investigated. Intrudingly, 200 *μ*M of H_2_S could completely abolish high glucose-induced ROS production, as determined by DCF fluorescence (Figures 6(a)–6(c)). Furthermore, 200 *μ*M of H_2_S could also completely abolish high glucose-induced NLRP3, ASC, and caspase-1 mRNA expression levels (Figures 6(d)–6(f)). Furthermore, H_2_S also led to the significant attenuation of proinflammatory cytokine mRNA expression and protein secretion (Figures 7(a)–7(d)).

## 4. Discussion

The present study is aimed at understanding the responses and mechanisms of human RPE cells, that is, the ARPE-19 cell line, under high-glucose conditions with or without extracellular H_2_S treatment. It was found that (1) high glucose could trigger a significant cell apoptosis and inflammatory response, as evidenced by the increase in ROS formation and NLRP3 inflammasome activation, (2) extracellular H_2_S addition could attenuate high glucose-induced cell apoptosis and inflammatory response, and (3) the protection provided by H_2_S was through blocking ROS formation and NLRP3 inflammasome activation.

Hyperglycemia has been implicated as an important contributing factor in DR progression through damaging the retinal microvasculature, resulting in retinal structure and function disorder [29]. In the present study, according to cell viability, inflammatory cytokines, intracellular ROS formation, and NLRP3 activation analysis, it was found that high glucose could significantly increase inflammation and apoptosis in ARPE-19 cells. A study conducted by Shen and Rong [29] indicated that mitochondrial ROS production was solely upstream responsible for high glucose-induced cell injury. Moreover, ROS has been considered to play a pivotal role in the activation of NLRP3 in the inflammasome, such as those reported in a rat model and HRMECs [14]. In 2002, the group of Prof. Tschopp described a multiprotein complex able to oligomerize and activate inflammatory caspases leading to the processing of IL-1*β* and IL-18. This complex was named NLR PYD-containing protein 3 (NLRP3) inflammasome [30]. In an excellent agreement, the present data revealed that hyperglycemia could induce the activation of the NLRP3 inflammasome and ultimately lead to the increased expression of IL-1*β* and IL-18. In addition, the silencing of NLRP3 gene expression through the siRNA approach could significantly ameliorate the high glucose-induced activation of IL-1*β* and IL-18. IL-1*β* is an inducible cytokine and is not generally expressed in healthy cells or tissue. Within the IL-1 cytokine family, IL-18 is most closely related to IL-1*β* and shares many common traits including cleavage by caspase-1 to a biologically active mature protein of ∼17 kDa that is actively secreted from cells. They all belong to the IL-1 family, which have been demonstrated to have broad and similar proinflammatory activity [31]. Taken together, these findings strongly support the important role of the NLPR3 inflammasome in high glucose-induced inflammation and apoptosis in RPE cells, as well as in the entire visual system.

At the same time, oxidative stress plays a pivotal role in the pathogenesis of diabetes and its complications [32]. ROS levels are markedly increased in various diabetic models and are parallel with enhanced cell injury. Devi et al. found that high glucose could induce more elevated oxidative stress and the apoptosis of retinal pericytes and that antioxidant NAC and azaserine could significantly attenuate high glucose-induced ROS formation and DNA damage [33]. Moreover, several studies have indicated that ROS could promote inflammation and cell apoptosis with the progression of DR [5, 34, 35]. Similarly, in the present study, it was also demonstrated that 25 mmol/L of NAC could almost abolish high glucose-stimulated NLRP3 inflammasome activation and downstream inflammatory factors. Collectively, these results suggest that ROS formation stimulated by high glucose could induce inflammation by activating the NLRP3 inflammasome in ARPE-19 cells.

Gasotransmitters are a group of gaseous molecules, with pleiotropic biological functions. These molecules include nitric oxide (NO), hydrogen sulfide (H_2_S), and carbon monoxide (CO). Abnormal production and metabolism of these molecules have been observed in several pathological conditions [36]. Among them, H_2_S has become recognized as an important signaling molecule throughout the body, contributing to many physiological and pathological processes [37], affecting the function and activity of intracellular and extracellular proteins through different metabolic pathways [38, 39]. The potential properties of H_2_S in improving pathological processes have been originally tested using H_2_S donors such as sodium hydrogen sulfide (NaHS), Na2S, N-acetylcysteine, or Lawesson's reagent. It has been shown that high concentrations of the H_2_S donor NaHS promote the release of TNF-*α* and IL-1 from IFN-*γ*-stimulated U937 cells, in an NF*κ*B-dependent manner. Furthermore, studies have suggested that it could cope with ischemic injury, elevated oxidative stress, cell apoptosis, and inflammation [40]. For instance, it has been found that H_2_S plays an important role in the regulation of pancreatic beta cell function, insulin resistance, and diabetes complications [25]. Furthermore, it can lower oxidative stress and various markers of vascular inflammation in diabetes [41]. A recent study conducted by Zhou et al. demonstrated that H_2_S could block diabetic nephropathy in a streptozotocin-induced diabetic rat experimental model via normalizing oxidative stress and inflammation, preventing mesangial cell proliferation, and inhibiting renin-angiotensin system activity [42]. In a model of streptozotocin-induced diabetes in rats, H_2_S formation was significantly increased in homogenates of the pancreas and liver of diabetic animals, as compared to healthy animals, and insulin treatment of streptozotocin-challenged rats reversed the increase in H_2_S-synthesizing activity [43].

RPE cells are part of the blood-retinal barrier and play a crucial role in the maintenance of the visual system. RPE cells can secrete PEDF, VEGF, and other inflammatory cytokines, which participate in numerous pathologic and physiologic processes [44]. Chronic hyperglycemia could trigger the damage of RPE cells, subsequently affect the blood-retinal barrier, and ultimately promote the pathogenic progression of DR [44]. Moreover, Parsanathan and Jain found that exogenous administration of NaHS, a H_2_S donor, can significantly upregulate the genes involved in GSH biosynthesis under diabetic conditions [45]. Furthermore, Jain et al. also showed that H_2_S upregulated the glutamate-cysteine ligase catalytic subunit (GCLC) and GSH in a monocyte cell exposed to high glucose levels [20]. Therefore, it could be speculated that H_2_S plays a protective role in the retina and DR by inhibiting ROS formation and inflammatory response at least partially through RPE cells, as reflected by a significantly increased level of GSH and reduced levels of proinflammatory cytokines IL-1*β* and IL-18, the decreased expression of NLRP3 inflammasome, and DCF fluorescence, which is a ROS marker. In addition, a significant reduction in IL-1*β* and IL-18 could be achieved after blocking either the ROS or the NLRP3 inflammasome complex, suggesting that the mechanism of H_2_S in attenuating high glucose-induced inflammation is mediated through the amelioration of the ROS-NLRP3 inflammasome pathway.

## 5. Conclusions

In conclusion, the present study revealed that high glucose-stimulated RPE cell IL-18 and IL-1*β* secretion was induced through the activation of NLRP3 and ROS production. H_2_S can reduce the expression of IL-18 and IL-1*β* in RPE cells likely *via* the inhibition of the ROS-inflammasome pathway. These findings may support the potential therapeutic role of H_2_S in DR.

## Figures and Tables

**Table 1:** Primers used for the PCR gene.

	Forward, 5′-3′	Reverse, 5′-3′
*β*-Actin	AGTGCCAGCCTCGTCTCATAG	CGTTGAACTTGCCGTGGGTAG
NLRP3	CATGAGTGCTGCTTCGACAT	GCTTCAGTCCCACACACAGA
ASC	GGCTGCTGGATGCTCTGTA	AGGCTGGTGTGAAACTGAAGA
Caspase-1	GCCTGTTCCTGTGATGTGGAG	TGCCCACAGACATTCATACAGTTTC
IL-18	GCATCAACTTTGTGGCAATGA	ATAGAGGCCGATTTCCTTGGT
IL-1*β*	TGGCAATGAGGATGACTTGT	TGGTGGTCGGAGATTCGTA

## Data Availability

All data generated or analyzed during the present study are included in this published article. More details are available from the corresponding author on reasonable request.

## Abbreviations

DR: Diabetic retinopathy
RPE: Retinal pigment epithelium
H_2_S: Hydrogen sulfide
ROS: Reactive oxygen species
NAC: Antioxidant N-acetylcysteine
PKC: Polyol pathway and protein kinase C
VEGF: Vascular endothelial growth factor
IGF-1: Insulin-like growth factor-1
AGEs: Advanced glycation end products
RAAS: Renin-angiotensin-aldosterone system
HRMECs: Human retinal microvascular endothelial cells
CSE: Cysteine by cystathionine gamma-lyase
GSH: Glutathione
H_2_O_2:_: Hydrogen peroxide
NaHS: Sodium hydrosulfide
NAC: N-Acetyl-L-cysteine
DCFH-DA: 2,7-dichlorofluorescein diacetate
CCK-8: Cell Counter Kit-8
DMEM/F12: Dulbecco's modified Eagle's medium F12
FBS: Fetal bovine serum
ATCC: American Type Culture Collection
DCF: Dichlorofluorescein
FACS: Fluorescence-activated cell sorting
PCR: Polymerase chain reaction
SE: Standard error
ANOVA: Analysis of variance
CSTC: Chongqing Key Laboratory of Ophthalmology.
